# A Rare Cause of Cyanosis: Hepatopulmonary Syndrome Caused by Congenital Extrahepatic Portosystemic Shunt

**DOI:** 10.1155/2011/508171

**Published:** 2011-12-20

**Authors:** Xue-Yan Ding, Feng Chen, Xian-Xian Zhao, Hong Wu, Shao-Ping Chen, Yong-Wen Qin

**Affiliations:** Department of Cardiology, Changhai Hospital, Second Military Medical University, Shanghai 200433, China

## Abstract

A 19-year-old male patient presented cyanosis and dyspnoea because of the presence of multiple pulmonary arteriovenous fistulas resulting in oxygen desaturation. The CTA revealed that intestinal and splenic venous blood bypasses the liver and drains into the inferior vena cava. This is the first reported case of hepatopulmonary syndrome caused by congenital extrahepatic portosystemic shunt in which intestinal and splenic venous blood bypasses the liver and drains into the inferior vena cava.

## 1. Introduction

Congenital extrahepatic portosystemic shunts are rare. They are often associated with other congenital anomalies, including biliary atresia, focal nodular hyperplasia of the liver, and polysplenia [1]. The complications are the following: hyperammonemia, encephalopathy, pulmonary arterial hypertension, and hepatopulmonary syndrome. The purpose of this report is to present a case of HPS associated with congenital extrahepatic portosystemic shunt in a 19-year-old male whose liver function and portal pressure is normal. Diagnostic challenge is discussed. 

## 2. Case Descriptions

**Figure 1:**
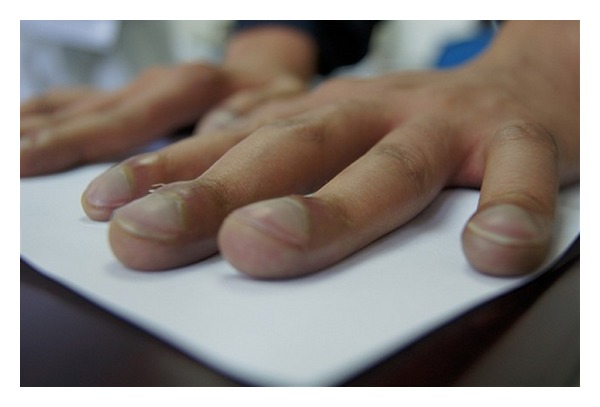
Finger clubbing.

**Figure 2:**
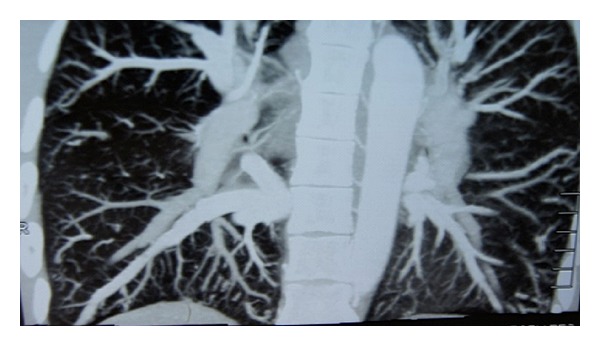
Pulmonary computed tomographic angiography (CTA) shows pulmonary arteriovenous malformation.

**Figure 3:**
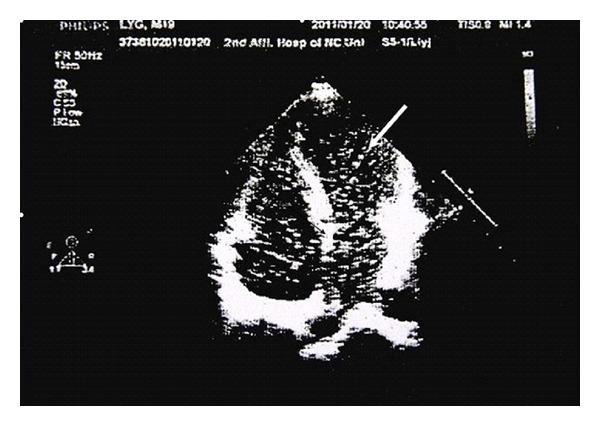
Contrast echocardiography shows that microbubbles (the arrow) produced by contrast agent enter the left ventricle three heart beats after the right.

**Figure 4:**
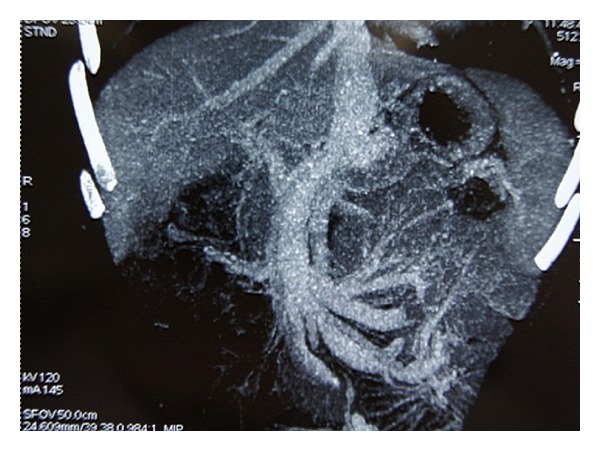
CTA reveals that intestinal and splenic venous blood bypassed the liver and drains into the inferior vena cava.

A 19-year-old male presented with a 2-year history of cyanosis and dyspnoea. He has never smoked and had no excessive alcohol, or any illicit drug, use. There was no familial history of congenital cardiovascular diseases. Physical examination on admission revealed cyanosis and finger clubbing (Figure 1). There were no murmurs or extra heart sounds. His partial pressure of oxygen was 49 mmHg. Laboratory studies reported that total bilirubin, aspartate aminotransferase, alanine aminotransferase, c-glutamyl transferase, ammonia, a-fetoprotein, albumin, total proteins, INR, are normal, but the hemoglobin level was 188 g/L. Chest radiography showed increased, diffused interstitial markings. No cardiac abnormality detected by cardiac catheterization, and all of these failed to diagnose specific cardiac defects of right-to-left shunts. Then, pulmonary computed tomographic angiography (CTA) shows pulmonary arteriovenous malformation, perhaps diffuse pulmonary arteriovenous fistulae (Figure 2). Contrast echocardiography then showed enhancement in the left heart three beats after microbubbles opacified the right heart, which confirmed intra-pulmonary shunting (Figure 3). This finding prompted us to survey his abdomen. The CTA revealed that intestinal and splenic venous blood bypasses the liver and drains into the inferior vena cava (Figure 4). No evidence of chronic liver disease was noted and there was no sign of hepatic encephalopathy. On the basis of these findings, he was diagnosed with congenital extrahepatic portosystemic shunt (Abernethy malformation), which may cause hepatopulmonary syndrome. 

## 3. Comments

Congenital extrahepatic portosystemic shunt was a rare malformation which was originally described by Abernethy in 1793 [2]. Morgan and Superina introduced a classification system based on whether the portal vein was present and whether the liver was perfused with blood from the mesenteric venous system [3]. In type I, portal blood is completely diverted into the vena cava with congenital absence of the portal vein. Type II shunts are partial shunts with portal-hepatic anastomoses. Congenital extrahepatic portosystemic shunt was often associated with other congenital anomalies, including biliary atresia, focal nodular hyperplasia of the liver, polysplenia. The complications of congenital extrahepatic portosystemic shunt are the following: hyperammonemia, encephalopathy, Pulmonary arterial hypertension (PAH), and hepatopulmonary syndrome (HPS). Our case belongs to type II without other congenital anomalies. Our patient did not present with hepatic encephalopathy which was common in patients with portal-systemic shunt, but with HPS. So far, there were only 3 case reports, in which congenital extrahepatic portosystemic shunt type II caused HPS [4–6]. 

Hepatopulmonary syndrome is characterized by intrapulmonary vascular dilatations causing right-to-left shunting, which leads to a range of arterial oxygenation abnormalities in patients with liver disease [7]. HPS may occur if portocaval shunting (associated or not with portal hypertension) affecting the metabolism of vasoactive substances by the liver. A definite diagnosis of hepatopulmonary syndrome requires scintigraphy with technetium-99 m-labelled macroaggregated albumin, pulmonary angiography, or contrast echocardiography. The pathogenesis of hepatopulmonary syndrome in the patient with congenital extrahepatic portosystemic shunt is not clear. The physiologic mechanisms commonly used to explain why HPS occurs in patients with impaired liver function include the following. First, elevated ET-1 circulating in the whole body may cause HPS [8]. Second, some hepatic products, which are necessary for pulmonary vasomotor control, are decreased by liver dysfunction or hepatic venous flow reduction [9, 10]. Third, translocation of gut bacteria causes accumulation and activation of macrophages in the lungs, which results in increase of inducible NO synthase production leading to vasodilatation [11]. Perhaps the mechanism is that the shunt impairs the metabolism of vasoactive pulmonary substances by the liver as the cause of hepatopulmonary syndrome in patients with portal hypertension.

Diagnosis of congenital extrahepatic portosystemic shunt is a challenge for clinicians, because most of patients present with unusual symptoms such as hepatic encephalopathy, PAH, HPS, in the absence of liver disease. In our case, the young patient presented with cyanosis and dyspnoea, and laboratory studies were normal except hemoglobin level. These symptoms usually make us take the common causes of dyspnea into consideration, such as COPD, specific cardiac defects of right-to-left shunts, asthma, and CHF. As HPS, it mostly occurs in patients with liver disease (associated or not with portal hypertension), but liver function of our patient was intact with normal histology, and portal pressure was normal. The pulmonary arteriovenous malformation promotes us to check his abdomen and then comfirm the diagnosis.

Treatment of congenital extrahepatic portosystemic shunt depends on the type, symptoms, and complications. In patients with type II Abernethy malformations, surgical ligation of the fistula can achieve regression of HPS provided that the hepatic portal system is sufficient to avoid portal hypertension.
